# Melatonin Improves Parthenogenetic Development of Vitrified–Warmed Mouse Oocytes Potentially by Promoting G1/S Cell Cycle Progression

**DOI:** 10.3390/ijms19124029

**Published:** 2018-12-13

**Authors:** Bo Pan, Haoxuan Yang, Zhenzheng Wu, Izhar Hyder Qazi, Guoshi Liu, Hongbing Han, Qingyong Meng, Guangbin Zhou

**Affiliations:** 1 Farm Animal Genetic Resources Exploration and Innovation Key Laboratory of Sichuan Province, College of Animal Science and Technology, Sichuan Agricultural University, Chengdu-611130, China; bopan1992@163.com (B.P.); yanghaoxuan940712@gmail.com (H.Y.); WZZ15680826096@163.com (Z.W.); vetdr_izhar@yahoo.com (I.H.Q.); 2 Department of Veterinary Anatomy & Histology, Shaheed Benazir Bhutto University of Veterinary and Animal Sciences, Sakrand-67210, Sindh, Pakistan; 3 National Engineering Laboratory for Animal Breeding, Key Laboratory of Animal Genetics and Breeding of the Ministry of Agriculture, Beijing Key Laboratory for Animal Genetic Improvement, College of Animal Science and Technology, China Agricultural University, Beijing 100193, China; gshliu@cau.edu (G.L.); hanhongbing@cau.edu.cn (H.H.); 4 State Key Laboratory of AgroBiotechnology, China Agricultural University, Beijing 100193, China; qymeng@cau.edu.cn

**Keywords:** melatonin, oocyte vitrification, redox homeostasis, cell cycle, developmental potential, mouse

## Abstract

This study aimed to investigate the effect of melatonin on the cell cycle of parthenogenetic embryos derived from vitrified mouse metaphase II (MII) oocytes. Fresh oocytes were randomly allocated into three groups: untreated (control), or vitrified by the open-pulled straw method without (Vitrification group) or with melatonin (MT) supplementation (Vitrification + MT group). After warming, oocytes were parthenogenetically activated and cultured in vitro, then the percentage of embryos in the G1/S phase, the levels of reactive oxygen species (ROS) and glutathione (GSH), and the mRNA expression of cell cycle-related genes (*P53*, *P21* and *E2F1*) in zygotes and their subsequent developmental potential in vitro were evaluated. The results showed that the vitrification/warming procedures significantly decreased the frequency of the S phase, markedly increased ROS and GSH levels and the expression of *P53* and *P21* genes, and decreased *E2F1* expression in zygotes at the G1 stage and their subsequent development into 2-cell and blastocyst stage embryos. However, when 10^−9^ mol/L MT was administered for the whole duration of the experiment, the frequency of the S phase in zygotes was significantly increased, while the other indicators were also significantly improved and almost recovered to the normal levels shown in the control. Thus, MT might promote G1-to-S progression via regulation of ROS, GSH and cell cycle-related genes, potentially increasing the parthenogenetic development ability of vitrified–warmed mouse oocytes.

## 1. Introduction

Oocyte cryopreservation, an adjunct to artificial assisted reproductive technologies, has been widely applied in the fields of medicine, agriculture and scientific research [1,2,3,4]. Especially in the medical field, it has provided the means for women suffering from ovarian cancer [5] or premature ovarian failure [6] and those planning to delay pregnancy [7] to reach their goals of having a baby. Moreover, it also has offered a convenient way to protect endangered wildlife germplasm resources [8] and to build superior breeding pools for livestock [9,10]. However, the survival rate of oocytes and their subsequent developmental competence are decreased significantly after vitrification when compared with these characteristics of fresh oocytes [11,12,13]. The decreased developmental potential due to oocyte cryopreservation may inevitably result from the alteration of intracellular levels of reactive oxygen species (ROS) [14] or glutathione (GSH) [15], and/or gene expression [16,17,18].

Reactive oxygen species, generated as a part of normal cellular metabolism, are essential for cell signal transduction [19,20]. At moderate levels, ROS produce beneficial effects on cellular responses and function. However, at higher concentrations, they can lead to severe detrimental effects such as DNA damage, lipid peroxidation and protein oxidation [21,22,23]. Glutathione, a small peptide molecule composed of only three amino acids, acts as an effective antioxidant and free radical scavenger and plays a key role in regulating cellular redox homeostasis [24]. Therefore, ROS and GSH are pivotal to maintaining the level of reductants and oxidants in a balanced state [25] in order to regulate oocyte maturation and normal development of zygotes. After oocytes are subjected to vitrification and warming, ROS levels are generally increased [13,14] and, conversely, GSH levels tend to decline [15,26,27]. In such situations, redox homeostasis would be perturbed, potentially weakening the quality of oocytes and reducing their developmental competence [28]. Moreover, under conditions of increased ROS levels, cell cycle progression during the in vitro development of mammalian oocytes and embryos is thought to be delayed or arrested [29,30]. Therefore, taking the foregoing facts into consideration, it is worthwhile to further elucidate how ROS and GSH levels are altered when oocytes are subjected to the vigorous procedures of cryopreservation and whether these induced changes affect the transition of cell cycle progression in parthenogenetic zygotes derived from vitrified–warmed mouse oocytes.

Similarly, the mRNA expression of stress-related genes (*Hsp70*, *Sod1*) [31,32], antioxidant genes (*MnSOD*, *CuSOD*) [33] and apoptosis-related genes (*P53*, *BCL2*, *BAX*) [34,35] would be altered after oocytes or embryos are subjected to vitrification-warming procedures. However, there is a comparative dearth of scientific evidence reporting potential impacts of the vigorous preconditions of cryopreservation on mRNA expression of cell cycle-related genes (*P53*, *P21* and *E2F1)*. *P53* and *P21* are core genes of cell cycle checkpoints and play a major role in maintaining cell cycle arrest [36,37]. In human cancer cells, G1 arrest was completely abrogated when P21 was deficient [38], and P53 expression overcame P21WAF1/CIP1-mediated G1 arrest and induced apoptosis [39]. E2F1 works as a transcription factor and is closely related to the G1/S transition and DNA synthesis [40]. Under conditions of reduced mRNA expression and reduced or inhibited activity of E2F1, essential components required for DNA replication are also substantially reduced and may lead to deficient or complete arrest in the G1/S transition [41,42]. Therefore, in view of the paucity of relevant reports on this particular topic, much remains to be elucidated. Hence, it is reasonable to study and explore whether the decrease in developmental competence of vitrified–warmed mouse oocytes is related to changes in the expression of cell cycle-related genes (*P53*, *P21* and *E2F1*) of the G1 phase.

Melatonin (MT), a scavenger of ROS, can promote oocyte maturation in vitro and enhance the rate of blastocyst formation of embryos cultured in vitro [43,44,45]. In this regard, in our previous study, we reported that 10^−9^ mol/L MT supplementation during vitrification/warming, activation and in vitro culture could increase the development potential of vitrified–warmed mouse metaphase II (MII) oocytes [13]. Meanwhile, previously MT was found to play an important role in cancer treatment. It could inhibit the proliferation of oncocytes by regulating cell cycle arrest and apoptosis [46]. Currently, it is largely unclear whether MT promotes the development potential of vitrified–warmed mouse oocytes by regulating redox homeostasis and cell cycle progression. 

The mouse has been regarded as a robust model for studying the mammalian embryonic development. Moreover, the extensive genome similarities between mouse and human being along with the experimental tractability of the mouse also furnish significant benefits to using this species. Moreover, mouse oocytes and embryos are also more readily available compared to those of other agriculturally-important livestock species [47]. Due to the practical, ethical and legal limitations, it is relatively difficult to use a human model of oocyte cryopreservation and embryo development in vitro in experimental studies requiring a higher number of oocytes and embryos. Thus, in the present study, we used a mouse model to elucidate the potential underlying mechanism of MT in promoting development of vitrified–warmed mouse oocytes in vitro by regulating cell cycle progression, cell cycle-related genes and redox homeostasis of parthenogenetic zygotes.

## 2. Results

### 2.1. Melatonin Promotes the G1/S Transition of Parthenogenetic Zygotes Derived from Vitrified Mouse Oocytes

**Figure 1:**
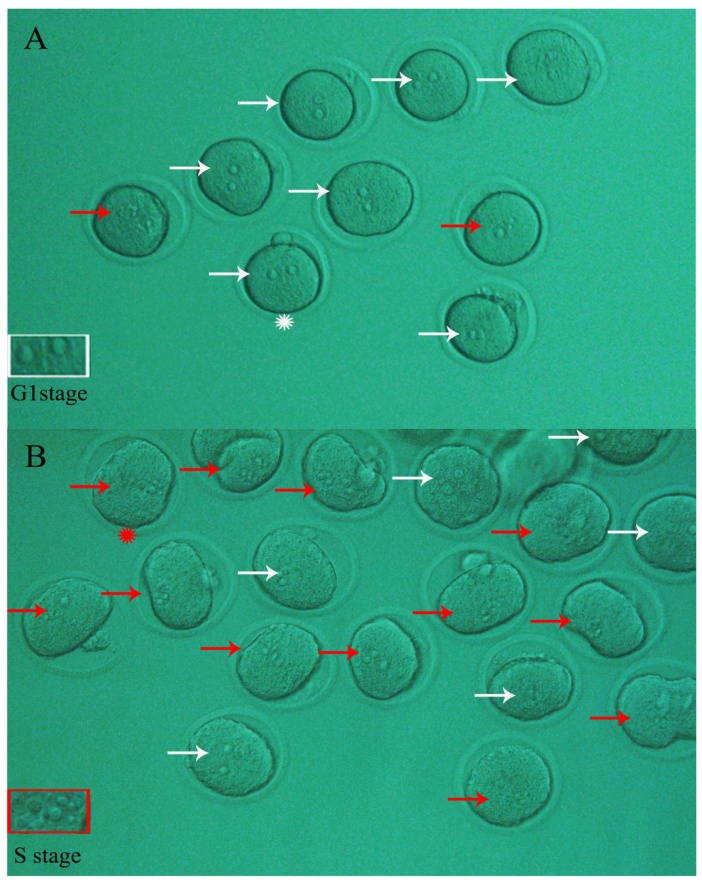
Typical phase showing nucleolus status of parthenogenetic zygotes. After parthenogenetic activation of mouse MII oocytes followed by in vitro culture for 3 to 4 h, the resulting zygotes ((**A**) 3 h; (**B**) 4 h) were observed under a stereomicroscope for determination of their nucleolus status. The embryos with two separate pronuclei and no apparent nucleoli inside (white arrows) remained at the G1 stage, while those with apparent nucleoli (red arrows) had proceeded through the G1 into the S phase. In Figure 1A, the criterion of pronuclei of parthenogenetic zygote in G1 stage is shown in the white rectangle (zoomed-in frame of corresponding zygote with white asterisk). Similarly, in Figure 1B, the criterion of pronuclei of parthenogenetic zygote in the S stage is shown in the red rectangle (zoomed-in frame of corresponding zygote with red asterisk). Original magnification 200×.

As shown in Figure 1A, a space was observed between two pronuclei and no nucleolus was evident in mouse zygotes (G1 stage) derived from activated MII oocytes followed by 3 h of in vitro culture. At this time point, more than 99.0% of the zygotes were observed at the G1 phase. When the activated oocytes were cultured in vitro for 4 h, 49.15% of the resulting zygotes proceeded to the S phase, as manifested by the appearance of nucleoli (Figure 1B). As shown in Table 1, the percentage of activated oocytes that developed to zygotes (S stage) in the Vitrification group was significantly lower (*p* < 0.05) compared to the Control (27.09% vs. 49.15%, respectively), indicating that the progression of the G1 into the S phase in embryos derived from vitrified oocytes was retarded. When 10^−9^ mol/L MT was added to the culture media used in the entire experiment, the percentage of embryos in the Vitrification group was significantly increased, from 27.09% to 40.42% (*p* < 0.05), suggesting that the G1/S transition progression in zygotes was accelerated.

### 2.2. Melatonin Decreased ROS Levels in Parthenogenetic Zygotes from Vitrified Oocytes

**Figure 2:**
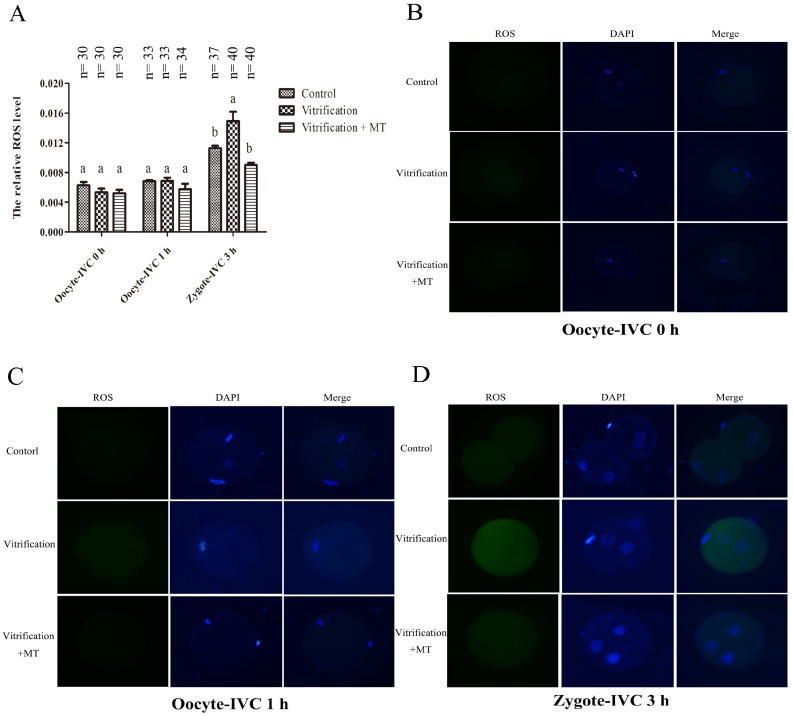
Reactive oxygen species (ROS) levels in mouse MII oocytes and their parthenogenetic zygotes. The dynamic change of ROS levels in mouse oocytes and their parthenogenetic zygotes (**A**) ROS staining in oocytes (**B**,**C**) and their parthenogenetic zygotes (**D**) Fluorescence intensities were correlated with intracellular levels of ROS. Different superscripts (a and b) represent treatment differences within panels (*p* < 0.05). After warming, mouse MII oocytes were in vitro cultured for 0 h (Oocyte-IVC 0 h) or 1 h (Oocyte-IVC 1 h) in M2 medium. The oocytes cultured for 1 h were selected for parthenogenetic activation (PA). During the entire experiment, all the media were supplemented with 10^−9^ mol/L (Vitrification + MT group) or 0 mol/L melatonin (Vitrification group). Fresh oocytes without melatonin (MT) treatment were used as controls (Control group). After PA and in vitro culture for 3 h, the resulting zygotes (zygote-IVC 3 h) were used for ROS detection together with mouse oocytes before PA. The values (the relative ROS levels)) are shown as mean ± SEM. The experiment was replicated at least three times. Original magnification 200×.

As shown in Figure 2A, there was no significant difference in ROS levels of mouse MII oocytes when they were cultured in vitro for 0 and 1 h among the Control, Vitrification and Vitrification + MT groups (*p* > 0.05). When activated oocytes were cultured for 3 h, the resulting zygotes (G1 stage) showed higher ROS levels in the Vitrification group than those of the Control group (*p* < 0.05). However, when MT was added to the medium, the ROS level decreased significantly and was similar to that of Control group (*p* > 0.05).

### 2.3. Melatonin Decreased GSH Levels in Parthenogenetic Zygotes from Vitrified Oocytes

**Figure 3:**
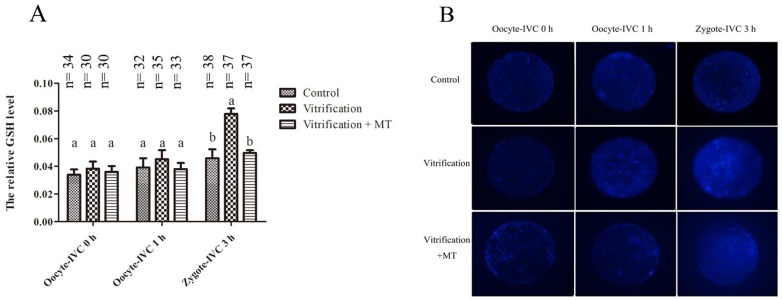
Glutathione (GSH) levels in mouse MII oocytes and their parthenogenetic zygotes. (**A**) The dynamic change of GSH levels in mouse oocytes and their parthenogenetic zygotes. (**B**) GSH staining of oocytes and their parthenogenetic zygotes in three groups. Fluorescence intensities were correlated with intracellular levels of GSH. The values (the relative GSH levels) are shown as mean ± SEM. The experiment was replicated at least three times. Different superscripts (a and b) represent treatment differences within panels (*p* < 0.05). Original magnification 200×.

As shown in Figure 3A, GSH levels in mouse MII oocytes that were cultured in vitro for either 0 or 1 h were similar among the Control, Vitrification and Vitrification + MT groups. However, after oocyte activation and 3 h of in vitro culture, the GSH levels in parthenogenetic zygotes were significantly higher (*p* < 0.05) in the Vitrification group than in the Control group. Supplementation of culture media with MT decreased GSH levels. Following MT supplementation in the culture media, GSH levels were decreased in the Vitrification + MT group and were comparable to the Control group.

### 2.4. Melatonin Altered mRNA Expression of G1 Checkpoint Related Genes in Parthenogenetic Zygotes from Vitrified Oocytes

**Figure 4:**
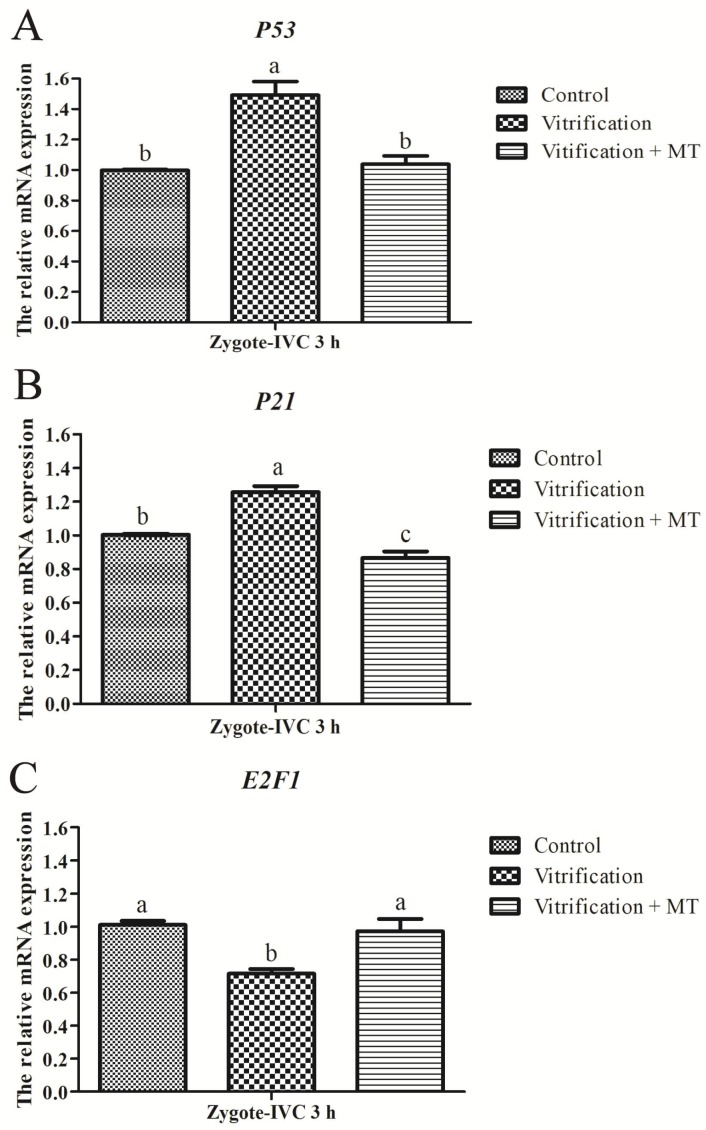
Effect of melatonin on mRNA expression of cell cycle-related genes in parthenogenetic zygotes (G1 stage). (**A**–**C**) The relative mRNA expression of cell cycle-related genes (*P53*, *P21* and *E2F1*) in zygotes at G1 stage. The relative expression levels of mRNA were determined by the 2^−△△*C*t^ method and normalized against that of the reference gene *GAPDH* (glyceraldehyde 3-phosphate dehydrogenase). All data are mean ± SEM from three replicates. Different superscripts (a, b and c) represent treatment differences within panels (*p* < 0.05).

Parthenogenetic mouse zygotes at the G1 phase were used for the detection of the expression of cell cycle-related genes (*P53*, *P21* and *E2F1*). As shown in Figure 4A, the mRNA expression of gene *P53* in the Vitrification group was significantly higher than that of the Control group (*p* < 0.05). When 10^−9^ mol/L MT was added to the media, the *P53* expression in the Vitrification + MT group decreased significantly to the level of the Control group. The *P21* expression in the Vitrification group was also significantly higher (*p* < 0.05) than in the Control group, and it was markedly decreased after the addition of MT to the culture media (Figure 4B). However, *E2F1* expression in the Vitrification group was significantly decreased (*p* < 0.05) when compared with the Control group, and after MT supplementation, it was markedly increased and was similar to that of the Control (Figure 4C). Alterations in the mRNA expression of cell cycle-related genes (*P53*, *P21* and *E2F1*) due to oocyte vitrification strongly suggested that cell cycle could be arrested at the G1 phase.

### 2.5. Melatonin Improved Parthenogenetic Development of Vitrified-Warmed Mouse Oocytes

After mouse MII oocytes were parthenogenetically activated, the resulting embryos were cultured in KSOM-AA medium. As shown in Table 2, the percentages of activated mouse oocytes that developed into embryos at the 2-cell, 4-cell, morula, blastocyst and hatched blastocyst stages in the Vitrification group were significantly lower (*p* < 0.05) than those of the Control group. After supplementation with MT, the percentages were markedly increased and almost recovered to the levels seen in the Control group (except for hatched blastocysts).

## 3. Discussion

Over the past few decades, the success of oocyte vitrification has progressed rapidly; vitrified–warmed oocytes could support development to term of fertilized [48,49,50,51,52] and cloned [53,54] embryos. However, the frequencies of live offspring derived from vitrified–warmed oocytes are unsatisfactory, which may result from such oocyte damage as mitochondrial dysfunction [28,55], DNA damage [56], metabolic disorders [57], and alteration of gene expression [13] due to vitrification, substantially hindering their subsequent developmental potential. Here, this was also confirmed by the fact that the parthenogenetic development of mouse oocytes into blastocysts significantly decreased from 66.67% to 33.61% after vitrification. In the present study we tried to elucidate the underlying mechanism by which melatonin promotes the development of vitrified–warmed mouse oocytes in vitro potentially by regulating cell cycle progression, expression of cell cycle-related genes and redox homeostasis.

Generally, in the G1 stage, cells are in an active metabolic state, preparing for synthesis of proteins, RNA and pre-replication complexes needed for DNA replication [58]. At this stage, the G1 checkpoint scrutinizes whether the DNA is complete to ensure normal DNA replication [59]. In the event of an undesirable state for DNA replication, cell cycle progression will be delayed or arrested [60,61]. Evidently, in the present study, oocyte vitrification delayed or arrested the G1/S transition in parthenogenetic zygotes. However, when MT (10^−9^ mol/L) was added to vitrification/warming solutions, and the medium used for oocyte activation/embryonic development, the G1/S transition in zygotes was significantly increased, showing that there was less delay in cell cycle progression. In breast cancer, MT promotes cancer cell apoptosis by blocking the G1/S transition [62]. It seems that the contradictory effects of MT on the regulation of cell cycle progression may be related to the different cell types (embryo vs. breast cancer cells) and/or cellular physiological state (normal vs. pathological). Nevertheless, more studies are required to further elucidate the underlying mechanisms to understand this particular discrepancy.

In the present study, the transcript levels of *P53* in parthenogenetic zygotes derived from vitrified-warmed oocytes were significantly up-regulated at the G1 phase compared to the Control group. Increased expression of transcription factor P53 due to oocyte cryopreservation would promote the mRNA expression of *P21* via the P53-P21 pathway [36]. When transcription of P21, a cyclin-dependent kinase inhibitor, is increased, it might down-regulate the activity [63] or transcription levels [64] of the E2F transcription factor family including E2F1. The low activity and protein levels of E2F1 thus induce G1 arrest [40,65], which was manifested here by the decreased percentage of parthenogenetic zygotes with nucleoli (S phase) from 49.15% to 27.09%. When 10^−9^ mol/L MT was administered, the transcription level of *P53* was significantly decreased in the vitrification group and restored to the level of the Control group, thus promoting the G1/S transition in parthenogenetic zygotes through P53-P21-E2F1 pathway and improving their subsequent in vitro development.

The cellular redox balance is required for normal cellular metabolism. However, when oocytes underwent cryopreservation and/or in vitro culture, high levels of ROS production [66] cause an imbalance in the intracellular redox systems, potentially leading to cell apoptosis or dysfunction [67,68]. Therefore, it would be beneficial for subsequent development of oocytes to reduce the production of excessive ROS. In the present study, interestingly, at the beginning (0 or 1 h) of in vitro culture, the levels of both ROS and GSH in vitrified–warmed oocytes were not significantly different in all three groups. At this point, we assumed that intracellular organelles might be in a recovery state following cryopreservation and consequently may have lower metabolic levels. After oocyte activation and in vitro culture for 3h, the resulting zygotes in the G1 stage exhibited higher levels of ROS in the Vitrification group than in the Control. However, when MT was added to the vitrification group there was no increase in ROS levels, consistent with the improved in vitro development of parthenogenetic embryos. Similarly, GSH levels in parthenogenetic zygotes at G1 stage were also increased after oocyte vitrification, which occurred potentially in response to ROS generation [69]. Another possible reason for the increased GSH levels may result from the higher expression of glutathione reductase and glutathione synthetase due to oocyte vitrification and in vitro culture of embryos. The GSH levels also decreased back to normal levels when MT was administered. The exact mechanism by which MT decreased GSH levels remains to be further investigated.

Recently it has been reported that mitochondrial quiescence is an effective pathway to ameliorate mitochondrial ROS-induced (mROS) oxidative damage in oocytes during in vitro maturation (IVM). Recently, He and colleagues evaluated the mitochondrial activity and expression of mitochondrial DNA (mtDNA) in porcine oocytes following MT treatment. Enhanced IVM rate, lipid droplet (LD) accumulation as well as triglyceride content in porcine oocytes were observed following MT supplementation in IVM medium. Reduced mitochondrial markers, such as mitochondrial membrane potential, mitochondrial respiratory chain complex IV activity and mROS levels, showed implication of MT in inducing a decrease in the mitochondrial activity [70]. Nevertheless, despite these enticing findings, much remains to be elucidated with respect to the potential implication of MT in the mitochondrial function and subsequent impact on outcomes of IVM of oocytes and embryo development.

The developmental potential of oocytes is not only related to their accumulation of maternal stores [71], but also to redox homeostasis in oocytes and preimplantation embryos. In response to external stimuli and/or increased ROS levels appearing during in vitro culture, embryos may show retarded development [72]. Such a negative impact, however, could be alleviated by addition of MT to the culture medium [13,73]. In a previous report, we found that vitrification of mouse oocytes increased intracellular ROS levels, and disorganized the mRNA expression of maternal-to-zygotic transition related genes in parthenogenetic 2-cell embryos [13]. In the present study, we further examined ROS and GSH levels and the expression of cell cycle-related genes (*P53*, *P21* and *E2F1*) in parthenogenetic zygotes at the G1 stage, and found that the increased ROS levels caused by oocyte vitrification would induce the mRNA expression of *P53* [74], which thus promoted *P21* transcription [38]; this suggests that excessive ROS could delay the division of parthenogenetic zygotes at the G1/S transition phase via the P53-P21 pathway and inhibit their further development. With the addition of MT to the Vitrification group, there were much lower ROS levels, potentially promoting the in vitro development of parthenogenetic zygotes by accelerating the G1/S phase transition via the P53-P21 pathway.

## 4. Materials and Methods

Unless otherwise stated, all chemicals were purchased from Sigma-Aldrich (St. Louis, MO, USA). All animals were maintained and handled in accordance with the requirements of the animal ethical and welfare committee (AEWC) of Sichuan Agricultural University (approval code: AEWC2016, 6 January 2016).

### 4.1. Oocyte Collection

Outbred female ICR mice (Dashuo Company, Chengdu, China) aged 6 weeks were kept in autoclaved cages in a room under standard conditions of a 14:10 light/dark cycle (light on at 06:00). After two weeks of acclimation, female mice were induced to superovulate by an initial intraperitoneal injection of 5 IU equine chorionic gonadotropin (PMSG, NingBo second hormone factory, Ningbo, China), and 48 h later 5 IU human chorionic gonadotropin (hCG, NingBo second hormone factory, Ningbo, China) was injected to trigger ovulation. Cumulus-oocyte complexes were collected from oviducts 12–14 h after hCG treatment and recovered in M2 medium [75] supplemented with 3 mg/mL bovine serum albumin. Cumulus cells were dispersed with 300 IU/mL hyaluronidase and then washed a minimum of 3 times in M2 for the subsequent experiments. During the entire experiment, we collected more than 2000 mouse oocytes, and 1870 were selected for use.

### 4.2. Oocyte Vitrification and Warming

Open-pulled straws (OPS) were made according to the method described previously [76,77]. Briefly, the straws (250 mL; IMV, L′Aigle, France) were heat-softened and pulled manually to produce a straw approximately 3 cm in length, 0.10 mm inner diameter, and 0.15 mm outer diameter.

Oocytes were vitrified using an OPS method. They were first equilibrated in 10% ethylene glycol (EG) + 10% dimethyl sulfoxide (DMSO) for 30 s, then loaded into the narrow end of an OPS with EDFS30 solution consisting of Dulbecco’s Phosphate Buffered Saline (DPBS) medium containing 300 g/L Ficoll, 0.5 mol/L sucrose, and 20% fetal bovine serum (FBS), 15% (*v*/*v*) EG and 15% (*v*/*v*) DMSO, with exposure for 25 s. Finally, the straws containing oocytes (8 oocytes per OPS) were quickly plunged into liquid nitrogen.

For warming, oocytes were rinsed in 0.5 mol/L sucrose for 5 min, then washed 3 times in M2 medium and incubated in an incubator (Thermo Electron Corporation, Marietta, OH, USA) at 37.5 °C with 5% CO2 in air for 1 h in M2 medium. All manipulations were performed at 37 °C on a warming stage fixed onto the stereomicroscope stage, and the ambient atmosphere was air-conditioned at a temperature of 25 ± 0.5 °C.

Before vitrification, oocytes were pooled and randomly distributed to each group (Control, Vitrification and Vitrification + MT). In the Vitrification + MT group, all the media (10% EG + 10% DMSO, EDFS30, 0.5 mol/L sucrose and M2) were supplemented with 10^−9^ mol/L of MT, while the Control and Vitrification groups did not contain MT.

### 4.3. Oocyte Parthenogenetic Activation and Embryo Culture

All oocytes were allowed to recover in a CO_2_ incubator for 1 h before parthenogenetic activation. The activation medium was Ca^2+^-free human tubal fluid (HTF) [78] supplemented with 10 mmol/L SrCl_2_ and 2 μg/mL cytochalasin D [79]. After being washed 3 times in activation medium, oocytes were incubated first in activation medium for 2.5 h and then in regular HTF supplemented with 2 μg/mL cytochalasin D for 3.5 h at 37.5 °C in a CO_2_ incubator. Finally, oocytes were removed from the above media and cultured in KSOM-AA with D-Glucose and Phenol Red medium [80] (CAT# MR-121-D, Millipore, Temecula, CA, USA) for 120 h. Embryo development was assessed at 24, 48, 72, 96 and 120 h after the start of culture. All the media in this experimental procedure were supplemented with 10^−9^ mol/L of MT only in the vitrification + MT group.

### 4.4. Detection of Cell Cycle Progression

Cell cycle procession in mouse embryos was assessed according to the method described previously [81]. In brief, according to the developmental morphology of the one-cell embryo derived from fertilized oocyte in vivo: at G1 phase, 12–21 h post-hCG injection, a space is evident between two pronuclei; at S phase, 21–27 h, two pronuclei are located very close to each other with the appearance of nucleoli; at G2 phase, 27–30 h, the profile of two pronuclei disappears in one-cell embryo and large particle appears in cytoplasm; at M phase, 30–33 h, the cellular body elongates and cytoplasmic division appears in the one-cell embryo. Therefore, bearing in mind the foregoing descriptions, we observed the morphology of parthenogenetic zygotes under an inverted phase contrast microscope (IX70, Olympus, Tokyo, Japan). When there was a space between the two pronuclei and no nucleoli, zygotes were classified to be at the G1 phase, and when there were two pronuclei located very close to each other with the appearance of nucleoli, zygotes were classified to have proceeded into S phase.

### 4.5. Measurement of Intracellular ROS and GSH

Mouse MII oocytes and their parthenogenetic zygotes at G1 phase were collected to determine the intracellular ROS and GSH levels according to a previous report [82]. To measure intracellular ROS levels, more than 10 oocytes or embryos from each treatment group were incubated (in the dark) in M2 supplemented with 1 mmol/L 2, 7-dichlorodihydrofluorescein diacetate (H2DCFDA, Invitrogen, Carlsbad, CA, USA) for 20 min at 37 °C, washed 3 times in M2 medium containing 3mg/ml bovine serum albumin, and then placed into 6 μL droplets of fluorescent mounting medium with DAPI (Vector Laboratories Inc., Burlingame, CA, USA) on a slide, then covered with a cover slip. Fluorescence was measured under an epifluorescence microscope with a filter at 460-nm excitation, and fluorescence images were recorded as TIFF files using a camera (BX53, Olympus, Tokyo, Japan). The recorded fluorescence intensities were quantified using Image J software (version 1.48; National Institutes of Health, Bethesda, MD, USA) after deducting the background value. The level of GSH in each oocyte was measured with 10 μmol/L 4-chloromethyl-6.8-difluoro-7-hydroxycoumarin (Cell-Tracker Blue, Invitrogen, Carlsbad, CA, USA) with a filter at 370-nm excitation. The experimental procedure was the same as the ROS measurement described above. The experiment was replicated 3 times.

### 4.6. Quantitative Polymerase Chain Reaction (Q-PCR)

Total complementary DNA (cDNA) was isolated from 20–25 parthenogenetic zygotes at the G1 stage for each group by using TransScript-Uni Cell to cDNA Synthesis SuperMix for Q-PCR (TransGen Biotech, Beijing, China). A total of 195 zygotes (Control: *n* = 65; Vitrification: *n* = 65; Vitrification + MT: *n* = 65) were collected for the Q-PCR test. Then, the cDNA was quantified by Q-PCR using TransStart Tip Green qPCR SuperMix (TransGen Biotech, Beijing, China) on a CFX Connect Real-Time Detection System (Bio-Rad, Hercules, CA, USA) under standard conditions. The cycle threshold (Ct) value used to calculate the relative expression was the average of 3 replicates and was normalized against that of the reference gene (*GAPDH*). The primer information is summarized in Table 3. The mRNA expression levels were calculated using the 2^−△△*C*t^ method [83].

### 4.7. Experimental Design

**Figure 5:**
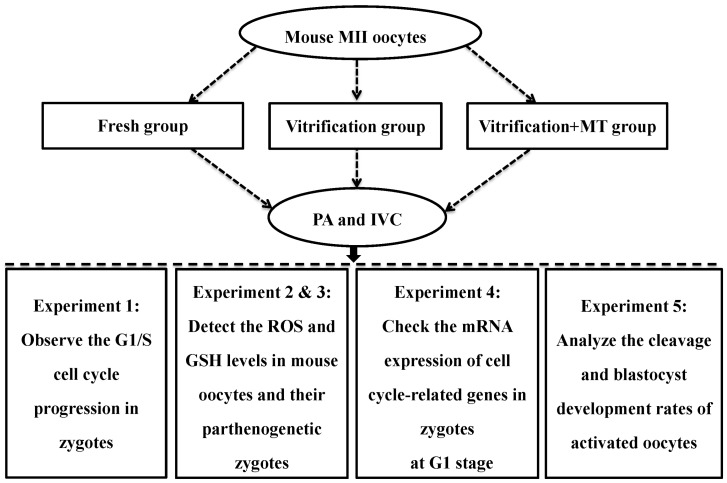
Flowchart of experimental design. Control group: untreated mouse MII oocytes; Vitrification group: oocytes were vitrified by the open-pulled straw method without melatonin (MT) addition; Vitrification + MT group: oocytes were treated with MT at a final concentration of 10^−9^ mol/L in all the media used in the entire experiment; PA: parthenogenetic activation; IVC: in vitro culture; MT: melatonin.

Based on our previous results [13], we selected 10^−9^ mol/L MT for the present study. The experimental design is shown in Figure 5. In experiment 1, the effect of MT was examined on cell cycle procession transition (G1/S) in parthenogenetic mouse zygotes. Mouse MII oocytes were first subjected to vitrification/warming and 1 h of in vitro culture, then to parthenogenetic activation (PA) followed by in vitro culture of the parthenogenetic embryos. The percentage of activated oocytes developing to parthenogenetic zygotes at the S stage was assessed to determine the effect of MT on the G1/S transition.

In experiments 2 and 3, the effect of MT on ROS and GSH levels in vitrified-warmed MII oocytes and their parthenogenetic zygotes was examined. Oocytes cultured for 0 or 1h in M2 medium and their parthenogenetic zygotes (G1 stage) were collected to detect ROS and GSH levels. We set the culture time (0, 1 and 3 h) based on the following considerations: (1) ROS and GSH levels in the oocytes were tested immediately (no culture, 0 h) after they were warmed, acting as a basal level. (2) When cultured in vitro for 1 h, the vitrified–warmed mouse oocytes almost recovered to a normal physiological state, which is suitable for PA. At this timepoint, we checked ROS and GSH concentrations to represent the normal physiological levels. (3) After PA and in vitro culture for 3 h, almost all the parthenogenetic zygotes were at the G1 stage. However, when the in vitro culture time was extended to 4 h, 49.15% of zygotes (Control group) proceeded to the S phase. Therefore, we selected parthenogenetic zygotes at the G1 stage (3 h) for assessing ROS and GSH levels.

In experiment 4, the effect of MT was investigated on cell cycle-related genes of parthenogenetic zygotes (G1 stage). The expression of these genes (*P53*, *P21* and *E2F1*) was determined by Q-PCR as described above.

In experiment 5, the effect of MT was tested on the in vitro development of parthenogenetic embryos derived from cryopreserved oocytes. The rates of cleavage (2-cells), and development to 4-cell embryos, morula, blastocyst and hatched blastocyst were assessed.

### 4.8. Statistical Analysis

All experiments were replicated at least 3 times. The percentages of activated oocytes that developed to zygotes in the G1/S phase and to subsequent embryos at the 2-cell, 4-cell, morula, blastocyst and hatched blastocyst stages were analyzed by the chi-squared test. Statistical analysis of ROS levels, GSH levels and gene expression was conducted by one-way ANOVA followed by the LSD test using SPSS (Version 20) statistical software (IBM, Chicago, IL, USA). Data were expressed as the mean ± standard error or mean ± standard deviation (SD), and *p* < 0.05 was considered statistically significant.

## 5. Conclusions

To sum up, mouse MII oocyte vitrification resulted in disturbances of the mRNA expression of cell cycle-related genes (*P53*, *P21* and *E2F1*), increased ROS and GSH levels in parthenogenetic zygotes, and decreased embryonic development in vitro after oocyte activation. Supplementation of media with 10^−9^ mol/L melatonin improved the in vitro development of parthenogenetic zygotes by regulating redox homeostasis (ROS/GSH) and the expression of cell cycle-related genes (*P53*, *P21* and *E2F1*).

## Figures and Tables

**Table 1:** Effect of melatonin on the G1/S transition in parthenogenetic zygotes.

Groups	No. of Oocytes Vitrified	No. of Oocytes Recovered	No. of Oocytes with Normal Morphology (%)	No. of Oocytes Activated	No. of Activated Oocytes Developed to	
					Zygotes in G1 Phase (%)	Zygotes in S Phase (%)
Control	-	126	126 (100 ± 0) ^a^	118	60 (50.85 ± 18.78) ^a^	58 (49.15 ± 18.78) ^a^
Vitrification	183	171	158 (87.68 ± 8.22) ^b^	155	113 (72.91 ± 10.89) ^b^	42 (27.09 ± 10.89) ^b^
Vitrification + MT	174	163	153 (89.59 ± 5.71) ^b^	141	84 (59.58 ± 8.74) ^a^	57 (40.42 ± 8.74) ^a^

Morphologically normal oocytes were evaluated by visual inspection of the membrane integrity, the zona pellucida (ZP), and any altered appearance of the cytoplasm (e.g., becoming white, colorless, or dispersed). The number of zygotes with nucleolus (S phase) or without nucleolus (G1 phase) was counted at 4 h after oocyte parthenogenetic activation (PA). Mouse MII oocytes from “Vitrification group” were first subjected to vitrification/warming and 1 h of in vitro culture, then to PA followed by in vitro culture of parthenogenetic embryos. During the whole experimental procedure, the other oocytes were treated either with 10^−9^ mol/L melatonin (MT) or without MT and vitrification/warming were classified as “Vitrification + MT group” and “Control group”, respectively. The experiment was replicated five times. The rate of oocytes with normal morphology (%) = (No. of oocytes with normal morphology/No. of oocytes recovered) × 100. The rate of zygotes in S stage (%) = (No. of zygotes in S stage/No. of oocytes activated) × 100. The values are shown as mean ± standard deviation (SD). Values with different superscripts (a and b) in the same column differ significantly (*p* < 0.05).

**Table 2:** Melatonin supplementation on parthenogenetic development of cryopreserved mouse MII oocytes.

Groups	No. of Oocytes Activated	No. of Activated Oocytes Developed to				
		2-Cell Embryos (%)	4-Cell Embryos (%)	Morula (%)	Blastocysts (%)	Hatched Blastocysts (%)
Control	150	141 (94.00 ± 2.55) ^a^	140 (93.33 ± 0.87) ^a^	129 (86.00 ± 1.94) ^a^	100 (66.67 ± 1.32) ^a^	50 (33.33 ± 15.35) ^a^
Vitrfication	122	90 (73.77 ± 11.96) ^b^	97 (79.51 ± 11.96) ^b^	80 (65.57 ± 12.32) ^b^	41 (33.61 ± 6.54) ^b^	8 (6.56 ± 4.78) ^c^
Vitrification + MT	175	160 (91.43 ± 9.62) ^a^	165 (94.29 ± 10.06) ^a^	147 (84.00 ± 7.72) ^a^	100 (57.14 ± 16.17) ^a^	39 (22.29 ± 3.82) ^b^

The rates of 2-cell, 4-cell, morula, blastocysts and hatched blastocysts were calculated from the total number of activated oocytes. For instance, the rate of 2-cell embryos (%) = (No. of zygotes cleaved/No. of oocytes activated) × 100. The values indicate the mean ± SD of five independent experiments. Values with different superscripts (a, b and c) in the same column are significantly different (*p* < 0.05).

**Table 3:** PCR primers used for SYBR green Q-PCR analysis.

Gene	Assay ID	Primer seq (5′-3′)	Product Length	Tm (°C)
*P53*	NM_001127233.1	F: AGGATTGTGGCCTTCTTTGA	126	62
		R: CAGATGCCGGTTCAGGTACT		
*P21*	NM_001111099.2	F: TGGAGATGAACTGGACAGCA	84	62
		R: TGAAGTTGCCATCAGCAAAC		
*E2F1*	NM_001291105.1	F: CGAGTCCTATGCCTTCAACA	159	62
		R: GAGTCCAGCCAGGAGATGAC		
*GAPDH*	NM_001289726.1	F: AGAACATCATCCCTGCATCC	124	62
		R: AGATCCACGACGGACACATT		

## Abbreviations

DMSO	dimethyl sulfoxide
DPBS	dulbecco’s phosphate buffered saline
DAPI	4′,6-diamidino-2-phenylindole
EG	ethylene glycol
FBS	fetal bovine serum
GAPDH	glyceraldehyde 3-phosphate dehydrogenase
GSH	glutathione
IVC	in vitro culture
IVM	in vitro maturation
LDs	lipid droplets
mtDNA	mitochondrial DNA
MII	metaphase II
MT	melatonin
OPS	open-pulled straws
PA	parthenogenetic activation
Q-PCR	quantitative polymerase chain reaction
ROS	reactive oxygen species
ZP	zona pellucida
